# Impact of age-friendly living environment and intrinsic capacity on functional ability in older adults: a cross-sectional study

**DOI:** 10.1186/s12877-023-04089-5

**Published:** 2023-06-17

**Authors:** Yi-Shan Jiang, Hong Shi, Yu-Ting Kang, Ji Shen, Jing Li, Ju Cui, Jing Pang, Chi Zhang, Jie Zhang

**Affiliations:** 1 School of Nursing, Chinese Academy of Medical Sciences and Peking Union Medical College, Beijing, China; 2 Department of Geriatric Medicine, Beijing Hospital, National Center of Gerontology, Institute of Geriatric Medicine, Chinese Academy of Medical Sciences, NO.1 Da HuaRoad, DongDan, Beijing, 100730 P. R. China; 3 Office of the National Clinical Research Center for Geriatric Diseases, Beijing Hospital, National Center of Gerontology, Institute of Geriatric Medicine, Chinese Academy of Medical Sciences, Beijing, P. R. China; 4 The Key Laboratory of Geriatrics, Beijing Institute of Geriatrics, Institute of Geriatric Medicine, Chinese Academy of Medical Sciences, Beijing Hospital/National Center of Gerontology of National Health Commission, Beijing, P. R. China

**Keywords:** Intrinsic capacity, Environment, Functional ability, Healthy aging

## Abstract

**Background:**

The World Health Organization (WHO) has proposed healthy aging framework, supposing that intrinsic capacity (IC), environment and their interaction may have influence on functional ability (FA). It was still unclear how the IC level and age-friendly living environment impact on FA. This study aims to confirm the relationship between the IC level and age-friendly living environment with FA, especially in older adults with low IC.

**Methods:**

Four hundred eighty-five community-dwelling residents aged ≥ 60 years were enrolled. IC constructed by locomotion, cognition, psychological, vitality, and sensory domains was assessed using full assessment tools recommended by WHO. Age-friendly living environment was measured with 12 questions adapted from the spatial indicators framework of age-friendly cities. FA was assessed using activities of daily living (ADL) and one question about mobile payment ability. Multivariate logistic regression was used to explore the association between IC, environment and FA. The influence of the environment on electronic payment and ADL under the IC layer was assessed.

**Results:**

Of 485 respondents, 89 (18.4%) had ADL impairment, and 166 (34.2%) had mobile payment function impairment. Limited IC (odds ratio [OR] = 0.783, 95% confidence interval [CI] = 0.621–0.988) and poor environment (OR = 0.839, 95% CI = 0.733–0.960) were associated with mobile payment ability impairment. Our results suggested that a supportive age-friendly living environment influenced FA was more prominent in older adults with poor IC (OR = 0.650, 95% CI = 0.491–0.861).

**Conclusions:**

Our results confirmed IC and the environment had an impact on mobile payment ability. The relationship between environment and FA showed differences according to IC level. These findings suggest that an age-friendly living environment is important to maintain and enhance elders’ FA, especially in those with poor IC.

**Supplementary Information:**

The online version contains supplementary material available at 10.1186/s12877-023-04089-5.

## Background

Coping with the trend that the aging population is rapidly accelerating worldwide, the World Health Organization (WHO) has released the *World report on ageing and health* and proposed the innovative concept of healthy aging to respond to the challenges of population aging. The report has set the ultimate goal of healthy aging to build and maintain functional ability that enables well-being in older age [1]. Functional ability (FA) comprises the health-related attributes enabling people to be and to do what they have a reason to value, and it is a final combination of the intrinsic capacity (IC) of individuals, the environments they inhabit, and the interaction between them [1].

WHO has defined IC as the composite of all the physical and mental capacities of an individual across their whole life [1], which is mainly constructed by five domains: cognition, locomotion, vitality, sensory (vision and hearing), and psychological [2]. These domains are related to the physical and psychological status, which are significant predictors of subsequent onset of disability and incident dependence [3–5]. Thus, it is considered that adverse FA may be potentially prevented by enhancing IC across the life [1]. WHO has offered care pathways to manage priority health conditions associated with a decline in IC and given recommendations on the assessment of IC at the community level [6]. However, very little evidence on the strict utility of the standard tools to measure IC is currently available. Since the majority of research comprises retrospective studies using collected data, the assessment of the sensory domain is limited and deficient [7]. Although some researchers use subjective inquiry to measure sensory domain [8], it is more inclined to increase social desirability bias and recall bias, less likely to reflect real capacity status [9].

In conformity with the healthy aging framework, the environment consists of all factors in the extrinsic world that form the context of an individual’s life, including home, communities, and the broader society [1]. Age-friendly cities and communities provide accessible and inclusive environments identified by eight topics [10]. Providing an age-friendly environment may make it easier for older adults to access services and facilitate their well-being [11]. Previous studies have identified that physical environmental factors, social network, and support may enhance or hinder functional ability [12]. Similarly, Lu et al. [13] have found that neighborhood physical environment shaped FA trajectories. Another study has investigated the relationship between environment and subjective well-being and found that the physical environment influenced FA more than the social environment [12]. However, little is known about the impact of age-friendly environment on FA. An understanding of the role of environmental factors may be central to strategies aimed at fostering health in older age.

As the healthy aging theoretical model hypotheses, IC is the main responsible factor for defining FA, in which an enabling environment provided across the elder’s life course might even increase IC and ultimately influence FA [1]. However, there is limited empirical evidence linking the interaction of environment and IC to impact the FA of the older persons. Moreover, it has been shown that mobile payment has digitized the payment method of silver generations’ day-to-day transactions, playing a critical role in assisting the senior population with their daily living. Especially during the coronavirus disease 2019 (COVID-19) pandemic, people have used mobile payment more frequently to reduce the chance of infection. Research has been focused on basic functional status, such as activities of daily life. However, to the best of our knowledge, no previous research has investigated mobile payment as FA, which is essential to meet older people’s needs. Therefore, this study aimed to measure IC more objectively within standardized integrated care for older people (ICOPE) tools and explore the impact of IC and age-friendly environments on the broad domains of FA.

## Methods

### Study design

This was a cross-sectional study conducted from December 2020 to January 2022 and no follow study were conducted. We conducted the study in the outpatient department of Beijing Hospital, which is located in the central urban area of Beijing. The major of senior citizens were recruited from communities surrounding the hospital from the main urban areas of Beijing, China. These communities have excellent infrastructure to meet the needs of mobile payment. Written informed consent was obtained from all respondents before inclusion. The study was approved by the Ethics Committee of Beijing Hospital (approval no.: 2020BJYYEC-240-02).

### Participants

Older people who aged ≥ 60 years and relatively healthy without acute infectious illnesses or progressive fatal diseases were included. Participants who could not cooperate in completing the survey due to various reasons (severe dementia, limited communication, language barriers, etc.), had a life expectancy of fewer than 6 months, or were participating in other clinic intervention trials were excluded. Data were collected via a face-to-face questionnaire interview and physical assessment by trained medical staff.

### Measures

#### Functional ability

FA was measured by the performance of activities of daily living (ADL), including basic activities of daily living (BADL) and instrumental activities of daily living (IADL), and one question about the adoption of mobile payment, representing a key domain of FA: meeting their basic needs. BADL was measured using the Katz index with six items including eating, dressing, getting in/out of bed, using the toilet, bathing and walking [14]. IADL was measured based on Lawton, with eight items of including preparing hot meals, taking medications, managing money, shopping for groceries, cleaning the house, and using the telephone [15]. The self-reported mobile payment adoption was assessed by asking the participants if they had problems using mobile payment services. For each BADL, IADL and mobile payment item, the correspondence was coded as 1 = no difficulty, 2 = some difficulty but can still do it, 3 = much difficulty and need help, and 4 = totally cannot do it. The total BADL and IADL score was 1 to 56, and the score of more than 1 was defined to be functional decline and 1 as no difficulties [16]. Participants with any self-reported impairment (score ≥ 1) in performing the mobile payment activity were defined as mobile payment function impairment.

#### Intrinsic capacity

IC is proposed to comprise five pivotal domains: locomotion, cognition, vitality, psychological, and sensory [2]. We used the following measurements to capture the multiple domains of IC under the recommendation of the WHO [6].

##### Locomotion

Locomotor capacity was measured by the Short Physical Performance Battery (SPPB). The internal consistency of SPPB, assessed as Cronbach’s alpha, was 0.76 [17]. The total scores of SPPB vary from 0 to 12 points, with 0–9 points representing limited mobility and 10–12 points representing normal mobility.

##### Cognition

The cognitive domain was assessed using the Chinese version Mini-Cog [18], the sensitivity and specificity of the Chinese version Mini-Cog has been verified to be 85.71% and 79.41% respectively [19]. Mini-Cog consists of two components: a delayed, three-word recall task and the clock drawing test. The three words recall scores of 0, 1, or 2 plus an abnormal clock drawing test represented positive for cognitive impairment.

##### Vitality

Vitality was assessed based on the Mini Nutritional Assessment Short-Form (MNA-SF), which has been widely used in China. MNA-SF has good internal consistency (alpha coefficient = 0.843) [20]. The MNA-SF score ranges from 0 to 14, and the score of 0–11 shows malnutrition or having the risk of malnutrition, which means vitality impairment, while the score of 12–14 demonstrates normal nutritional status [20].

##### Psychological

The psychological state was measured using the validated Chinese version of Patient Health Questionnaire (PHQ-9) to screen for depressive symptoms. Cronbach’s α coefficient of Chinese PHQ-9 was 0.833, and the test-retest coefficient reliability of PHQ-9 was 0.934 [21]. Each of the nine items can be scored from 0 (not at all) to 3 (nearly every day) to assess the severity of the symptoms [22]. The scores from 0 to 4 were considered a normal state, and scores from 5 to 27 were considered depression or psychological capacity impairment.

##### Hearing

Hearing was measured by the Whisper test according to the following procedures [6]: the evaluator sits about one arm’s length away behind and to one side of the participants, breathes out, and softly whispers four common unrelated words. If the person repeated more than three words and the researcher was sure they could hear clearly, then the person was likely to have normal hearing.

##### Vision

A WHO simple eye chart was used to measure the visual acuity of distance vision at three meters [6]. Participants were tested without glasses if normally worn. Visual acuity worse than 6/7.5 was considered a visual impairment.

For all elements of six IC aspects, the scores were re-scaled to a range of 0–1, with zero being the functional impairment and 1 being the normal function. Then, the IC summary score was added up, with a total score ranging from 0 to 6, where the higher scores indicated better performance.

### Age-friendly environment

The age-friendly environment was assessed with a set of 12 questions adapted from the spatial indicators framework of age-friendly cities [23]. The assessment tool was adapted from previous key papers [13, 24]. We conducted two rounds of expert validation of this questionnaire, the CVI of the age-friendly environment questionnaire was 0.943. Environments were assessed by indicators of availability of facilities (six items, example item: “Whether there were elevators in the community the older adults lived”), public transportation (“Whether there were bus or subway stations within 15 min’ walk”), healthcare services (three items, “Whether there were hospital or clinic within 15 min’ walking distance”), outdoor spaces and buildings (“Whether there were parks or greenfield within 15 min’ walk”), and social participation (“Whether there were senior centers within a 15 min’ walk”). Values ranged from 0 (without facility) to 1 (with facility) for each item. The sum of scores was calculated, with a total score ranged from 1 to 12, and we use the median as the cut-off value [25]. Component items are presented in Additional file 1.

### Covariates

The data on socioeconomic status, disease and medication situation were collected. Polypharmacy was defined as taking more than five drugs [26]. Multimorbidity was defined as participants self-reported of more than 2 chronic diseases diagnosed by a doctor [27]. Other sociodemographic characteristics included age, sex, marital status, educational level, monthly income. A college’s degree or above is considered to be a high level of education. Household income per capita of > 4,000 yuan was considered a high-income level.

### Statistical analysis

Data were presented as means ± standard deviation (SD) or median (inter-quartile range, IQR) for continuous variables and frequencies or percentages for categorical variables. multivariate logistic regression models were performed to evaluate the effect of IC and environment on functional status and e-payment as outcomes. We adjusted for age, sex, educational level, marriage status, and income level in Model 1 to evaluate the effect of potential confounders on this association. Furthermore, polypharmacy and multimorbidity were added in Model 2. Meanwhile, in order to further explore the role played by the environment under different IC level, we divided the participants into a better IC group and a worse IC group according to the median of IC score. To analyze the influence of environment on electronic payment and ADL, multivariate logistic regression was conducted under different IC layer. Co-linearity diagnosis is used to evaluate the existence of multilinearity between variables. All statistical analyses were performed using the Statistical Analysis Software (SAS) version 9.4 (Cary, NC, USA), and the level of statistical significance was set at *p* of < 0.05, two-tailed. Those subjects with any missing values were excluded for the analysis.

## Results

### Participant characteristics

A total of 485 participants with complete data were included, and 11 were excluded because of missing data. The average age of all participants was 69.88 ± 6.62 years, with most of them (60.6%) being female, 33.2% have a high level of education, 78.8% have a spouse, 43.9% of participants have a monthly income greater than 4,000 yuan. 116 (23.9%) of participants had polypharmacy and 72.6% were comorbidities.

The enrollers were more likely to belong to a normal cognition status (74.6%), have few depressive symptoms (16.7%), and have more visual impairment (64.3%) and less hearing impairment (2.7%). The mean SPPB and MNA-SF scores were 11.58 (SD = 1.18) and 13.08 (SD = 1.37), respectively, with 4.7% and 10.9% representing the impairment in each dimension. The average median score of IC and environment was 5 and 9, respectively. Among all participants, the proportion of impairment in ADL and mobile payment was 18.4% and 34.2%, respectively, with the incident of ADL disability for Katz and Lawton was 2.1% and 18.4% respectively. More than half (63.3%) reported good environment and 178 (36.7%) participants reported poor environment.

### Factors associated with functional ability

Multivariable logistic regression analysis indicated that participants with decreased ADL and mobile payment impairment were older (*P* < 0.001), less educated (*P* < 0.01), in a poor economic condition (*P* < 0.05), having a greater number of comorbidities (*P* < 0.01). However, no significant differences were observed in sex, marriage status or polypharmacy in terms of a greater deterioration of ADL or E-payment (*P* > 0.05) (Table 1).

**Table 1:** Multivariable logistic regression analysis for activities of daily living impairment and mobile payment impairment

Characteristic	Sub-categories	ADL Impairment			Mobile Payment Impairment		
		*OR*	*95%CI*	*P* Value*	*OR*	*95%CI*	*P* Value*
Age		1.103	1.060–1.149	** < 0.001**	1.153	1.108–1.199	** < 0.001**
Gender	Male	Ref			Ref		
	Female	0.777	0.445–1.355	0.374	0.969	0.605–1.553	0.896
Educational level	College’s degree below	Ref			Ref		
	College’s degree or above	0.450	0.246–0.825	**0.010**	0.346	0.207–0.581	** < 0.001**
Marriage status	Without a spouse	Ref			Ref		
	With a spouse	0.745	0.395–1.404	0.363	1.240	0.689–2.231	0.473
Income	Low income	Ref			Ref		
	High income	0.474	0.269–0.833	**0.010**	0.421	0.265–0.670	** < 0.001**
Polypharmacy	No	Ref			Ref		
	Yes	1.375	0.796–2.375	0.254	1.374	0.833–2.269	0.214
Comorbidity	No	Ref			Ref		
	Yes	3.879	1.569–9.589	**0.003**	2.920	1.577–5.406	** < 0.001**

Boldface indicates significance

*IC* Intrinsic capacity, *ADL* Activities of daily living

^*^Multivariable logistic regression

**Fig. 1:**
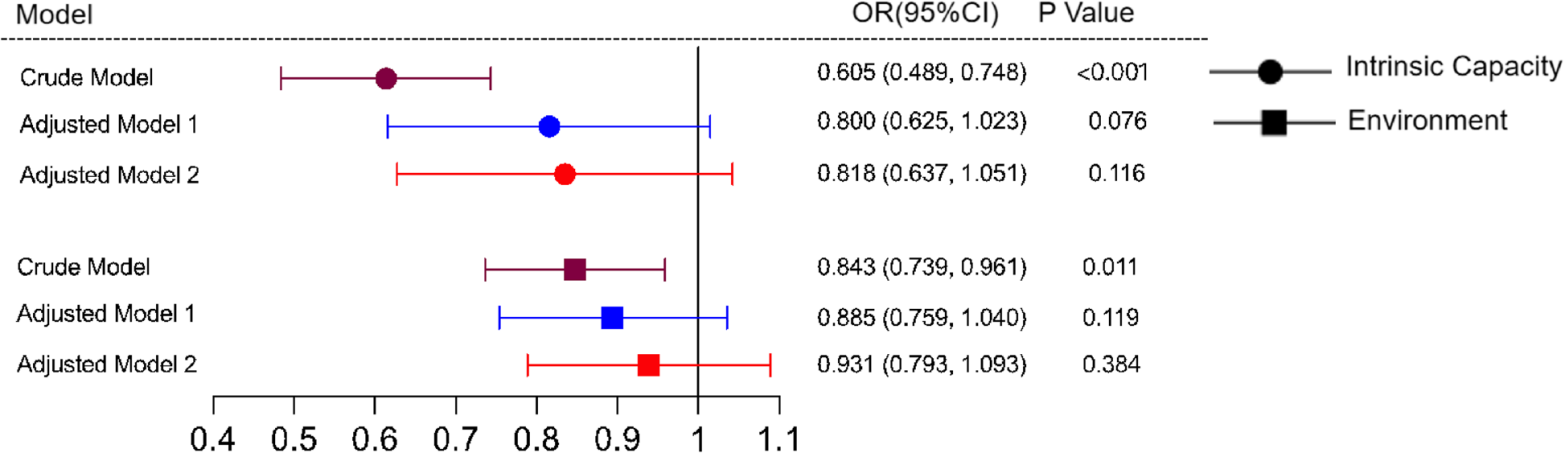
The Forest Plot of the Associations of Intrinsic Capacity and Environment With Activities of Daily Living Impairment. Adjusted model 1: adjustment for marriage status, educational level, age, sex, and salary income. Adjustment model 2: adjustment for polypharmacy and multimorbidity. *CI*, confidence interval

Three models were constructed to analyze the independent effect of IC and environment on the ADL and mobile payment through multivariate logistic regression. The variance inflation factor (VIF) among confounding factors was < 2 in all models, showing that there was no strong multicollinearity. In the model of intrinsic capacity and environment, VIF was 1.026 ~ 1.271 and 1.029 ~ 1.230, respectively. Although sex and marriage status did not show a significant difference in the multivariable logistic regression analysis, previous research has found that these two factors have an impact on the FA; hence, we adjusted them in model 1 [28]. In the unadjusted (crude) model, the impact of IC on ADL impairment was statistically significant (*P* < 0.001). However, in model 1 (adjusted for age, sex, marriage status, educational level, and income level) and model 2 (adjusted for the polypharmacy and multimorbidity), the association between IC and ADL was not stable (*P* > 0.05). Similar results were obtained for the environment (Fig. 1).

**Fig. 2:**
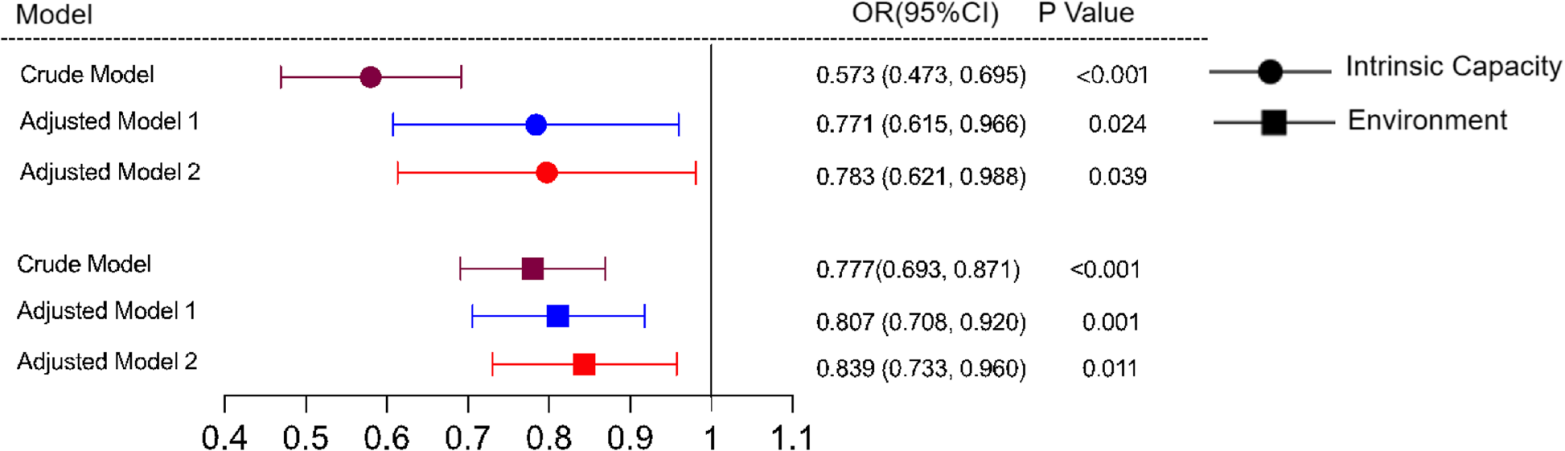
The Forest Plot of the Associations of Intrinsic Capacity and Environment With Mobile Payment Ability Impairment. Adjusted model 1: adjustment for marriage status, educational level, age, sex, and salary income. Adjustment model 2: adjustment for polypharmacy and multimorbidity. *CI*, confidence interval

Figure 2 illustrates the adjusted relationships between IC and environment with mobile payment. All regression analyses showed that a significant relationship existed between IC and mobile payment ability impairment after adjusting for demographic characteristics (*P* < 0.05). Our results also demonstrated that mobile payment ability was significantly influenced by the environment (*P* < 0.05) (Fig. 2).

### The effects of environment on functional ability under different level of intrinsic capacity

We stratified the level of IC to explore the impact of environment on FA. Participants was divided into subgroup according to their IC level (IC score of < 5 and ≥ 5), with the median 5 as the cut off point [25]. In the group of poor or good IC, VIF was 1.096 ~ 1.275 and 1.014 ~ 1.212 respectively. The results showed there was a significant relationship between environment and mobile payment in older adults with poor IC (*P* < 0.05). Moreover, among the older adults with good level of IC, there was a significant association between environment and impaired mobile payment ability in the crude model. After adjusted covariates, the environment just approached the borderline of significance (*P* = 0.061), indicating that the environment partially influenced mobile payment in older adults with good IC. However, an age-friendly living environment had little impact on ADL regardless of IC level (Table 2).

**Table 2:** Effects of environment on activities of daily living and e-payment in different intrinsic capacity groups

	Score of intrinsic capacity < 5		Score of intrinsic capacity ≥ 5	
	OR (95%CI)	*P*	OR (95%CI)	*P*
ADL				
Crude model	0.878 (0.705–1.092)	0.241	0.832 (0.700–0.989)	**0.037**
Model-1	0.900 (0.696–1.163)	0.420	0.889 (0.729–1.085)	0.246
Model-2	0.962 (0.731–1.264)	0.779	0.929 (0.755–1.142)	0.484
Mobile Payment				
Crude model	0.730 (0.591–0.901)	**0.003**	0.808 (0.702–0.930)	**0.003**
Model-1	0.637 (0.483–0.840)	**0.001**	0.863 (0.739–1.007)	0.061
Model-2	0.650 (0.491–0.861)	**0.003**	0.900 (0.767–1.056)	0.197

Crude model = environment

Adjusted model 1: adjustment for age, sex, educational level, marriage status, and salary income

Adjustment model 2: adjustment for polypharmacy and multimorbidity

## Discussion

This study was performed to identify the influence of IC and the environment and the impact of their interaction on FA. To the best of our knowledge, the present study is the first using mobile payment ability as one of FA measurements to investigate the impact of IC and age-friendly living environment on FA. The results showed empirical support for the construct of the WHO healthy aging framework [1], which emphasizes the effects of an age-friendly living environment on FA, especially in those with poor IC.

We found that participants with FA impairment were older. This result corresponds to previous studies wherein an increasing age has been significantly related to a decline in FA [29]. Furthermore, the study showed that lower education was associated with FA impairment; however, this is inconsistent with a previous study conducted in China [13]. Gender was not significantly associated with functional ability, which is consistent with previous studies [13, 30]. Additionally, the findings suggest that older adults with multimorbidity were more susceptible to less than optimum FA, consistent with another study [31]. Therefore, it is of great importance to control some specific chronic diseases and pay more attention to avoiding FA limitation in older adults.

We found that older persons with poor IC were more likely to have mobile payment capacity impairment. This might be explained by the fact that as individuals’ age, the declining abilities, such as physical, sensory, and cognitive ones, may lead to significant barriers in mobile use [32]. Our results also showed that age-friendly environment was positively correlated with digital payment ability in community-dwelling older adults. Lu et al. [13] reported the number of leisure and public transportation facilities was significantly associated with FA. The old generation desires to age in their community, service facilities and amenities in the neighborhood may underscore the urgent need for older adult residents’ to learn about technology use [33], influencing the acceptance of technology [34], facilitating and creating a social learning environment for older adults to perform everyday tasks, such as using mobile payment. Therefore, creating an age-friendly environment where older adults are actively involved, valued, and supported with infrastructure and services may effectively accommodate their needs [35].

No significant association was found between the overall IC and ADL. Investigations on functional ability in the old generation have shown conflicting results, with another study in China indicating that each IC domain was associated with ADL [30]. Some hypotheses for the differences may be related to that we used an integrative approach to summarize the IC levels. Although studying domain-by-domain associations with FA is essential, the construct of IC is whole by nature, and utilizing a holistic approach to IC may strengthen its validity [36].

We further explored how the intrinsic ability and environment interact to impact the FA of the older adults. We found that after adjusting covariates, the effect of environment on ADL was not significant regardless of IC level. This result indicated that age friendly environment might not be a salient factor in affecting the performance of fundamental self-care activity tasks. A previous systematic review has also found that physical and built neighborhoods generally demonstrate little impact on activity limitation [37].

However, multivariable logistic regression analysis showed that the significant effect of an age-friendly living environment on mobile payment remained after adjusting for variables in the lower IC group. According to a recent study, for older adults with a limited IC, the lack of access to important facilities is more likely to negatively impact their physical functioning [24]. The present study emphasized that an age-friendly living environment plays a different role depending on the level of intrinsic ability. Our findings support and verify the framework for *healthy aging* in that the role of the environment enabling FA will broaden as capacity declines. As decrements in a person’s intrinsic capacity increase, the benefits in FA accrued from the environment may become increasingly important [1]. Interventions targeting the creation of supportive and age-friendly environments need more emphasis; hence, the aged can preserve their functional capacity in advanced ages.

### Limitations and strengths

Our study has certain limitations. First, this study was a cross-sectional study, further research is needed to examine the longitudinal association of IC and environment with FA. Second, it was not a multicenter study, and we did not use random sampling method to recruit subjects, therefore, the representation of the population is limited. The subsequent research should be conducted with a multi-center and random sampling method. Third, we chose the basic elements and services of an age-friendly environment to design the environment scale; thus, further study should consider other indicators of age-friendly cities based on this scale to provide strengthened scientific evidence. Forth, the total score of IC was counted by each domain, and we used the median as the cut-off point for IC. A universal accepted cut-off point of IC is needed in future studies.

Despite these limitations, the study is unique in several aspects. Firstly, this study considered that in addition to traditional function, electronic payment ability of the older adults plays a vital role which affects their quality of life and cannot be ignored. Secondly, our study provides supplemental information on the effect of age-friendly environment on functional performance, future studies can further examine the longitudinal effects of age-friendly environment on the trajectory of functional decline.

## Conclusions and implications

The healthy aging framework emphasizes the key role of FA, and preserving and enhancing FA should be the ultimate goal of public health. Our study provided evidence for creating an age-friendly city and community environment to support older adults’ well-being and function. Additionally, our study verified the *healthy aging* framework and highlighted that interventions targeting the environment of the old population with declining IC need more emphasis.

## Supplementary Information

**Additional file 1.** The Age-friendly Environment Scale.

## Data Availability

The datasets used and analyzed during the current study are available from the corresponding author (Jie Zhang) on a reasonable request.

## Abbreviations

WHO: World Health Organization
IC: Intrinsic capacity
FA: Functional ability
ADL: Activities of daily living
ICOPE: Integrated care for older people
BADL: Basic activities of daily living
IADL: Instrumental activities of daily living
SPPB: The Short physical performance battery
MNA-SF: The Mini Nutritional Assessment Short-Form
PHQ-9: Patient Health Questionnaire
