# Supplementary material for: Impact of age-friendly living environment and intrinsic capacity on functional ability in older adults: a cross-sectional study
Source: BMC Geriatr. 2023 Jun 17;23:374. doi: 10.1186/s12877-023-04089-5 (PMC10276510; doi:10.1186/s12877-023-04089-5)
Supplement: Supplementary file 1 — Additional file 1. The Age-friendly Environment Scale. [file 12877_2023_4089_MOESM1_ESM.docx]

**The Age-friendly Environment Scale**

| Item | Response |
| --- | --- |
| 1. Are there any elevators in the residential area? | Yes (1) / No (0) |
| 2. Are there any barrier-free facilities in the residential area? | Yes (1) / No (0) |
| 3. Is there a bus/subway station within 15 minutes walk in the residential area? | Yes (1) / No (0) |
| 4. Is there a shopping mall/supermarket/wet market within 15 minutes walk in the residential area? | Yes (1) / No (0) |
| 5. Is there a restaurant/canteen within 15 minutes walk in the residential area? | Yes (1) / No (0) |
| 6. Is there a park/green space within 15 minutes walk in the residential area? | Yes (1) / No (0) |
| 7. Is there a senior center within 15 minutes walk in the residential area? | Yes (1) / No (0) |
| 8. Is there a hospital/clinic within 15 minutes walk in the residential area? | Yes (1) / No (0) |
| 9. Is there a pharmacy within 15 minutes walk in the residential area? | Yes (1) / No (0) |
| 10. Is there a day care centre/nursing home within 15 minutes walk in the residential area? | Yes (1) / No (0) |
| 11. Is there a bank within 15 minutes walk in the residential area? | Yes (1) / No (0) |
| 12. Is there a courier station within 15 minutes walk in the residential area? | Yes (1) / No (0) |
| **Total Score:** | |
